# Facilitators of Physical Activity: Voices of Adolescents in a Disadvantaged Community

**DOI:** 10.3390/ijerph14080839

**Published:** 2017-07-26

**Authors:** Linus Jonsson, Christina Berg, Christel Larsson, Peter Korp, Eva-Carin Lindgren

**Affiliations:** 1 Department of Food and Nutrition and Sport Science, University of Gothenburg, 41120 Gothenburg, Sweden; christina.berg@ped.gu.se (C.B.); christel.larsson@gu.se (C.L.); peter.korp@ped.gu.se (P.K.); eva-carin.lindgren@hh.se (E.-C.L.); 2 School of Health and Welfare, Halmstad University, 30118 Halmstad, Sweden

**Keywords:** child perspective, focus group interviews, low socioeconomic status, multicultural community, self-determination theory, youth

## Abstract

Despite increasing socioeconomic inequalities in the health and well-being of adolescents, the voices of adolescents in disadvantaged communities regarding facilitators of physical activity (PA) have received relatively little attention. In response, the purpose of this study was to illuminate what adolescents in a multicultural community of low socioeconomic status (SES) in Sweden convey concerning facilitators of PA. Adolescents (*n* = 53, aged 12–13 years) were recruited from a school in a multicultural community of low SES in Sweden. Following an interpretive approach, 10 focus group interviews were conducted to produce data for a qualitative content analysis. When the adolescents mentioned PA, they mostly referred to spontaneous PA rather than organized PA, and expressed that they enjoyed their PA engagement, which they stated was promoted by the variation of PA, available options for PA, their physical skills, and the presence of peers. They reported that social support from family and friends facilitated their PA, and they offered several suggestions regarding how the school environment could better support their PA. From the perspective of self-determination theory (SDT), the results stress the importance of facilitating intrinsic motivation with a supportive PA environment in which adolescents can satisfy their needs for autonomy, competence, and relatedness.

## 1. Introduction

There is a broad consensus that taking part in physical activity (PA) and sport from an early age is important for developing a foundation for lifelong physical engagement in healthy PA experiences [1,2]. In response, by listening to the perspectives of adolescents, some scholars have sought to gain insights into their views and experiences regarding PA, as well as to learn what could facilitate their PA [3,4]. By exploring these perspectives, scholars have identified several facilitators of PA among adolescents at the individual, social, environmental, and societal levels [3,4]. Most of these studies, however, have been conducted in the United Kingdom and the United States, and comparatively few have concentrated on adolescents in multicultural areas of low socioeconomic status (SES) [5,6,7,8]. Some facilitators of PA that seem to be specific to adolescents from such areas have been identified, however, including staying out of trouble [6] and the proximity of places of worship that provide social support [7].

Yet, earlier findings have to be understood within the context of the time and space in which they occurred [9]. During the last few years, a significant number of people have fled their homes for Europe due to persecution, military conflict, and poverty in their native lands [10]. Related to this great migration is increasing socioeconomic segregation throughout Europe, including in Sweden [11]. Regrettably, the attendant socioeconomic inequalities are also obvious in the health of adolescents [12], and adolescents from families of low SES engage in less moderate-to-vigorous PA, reporting poorer life satisfaction and self-rated health, than adolescents from families of high SES [13]. In Sweden, a recent study reported that only roughly 40% of boys and 20% of girls are physically active enough to fulfill current PA recommendations of 60 min of moderate-to-vigorous PA every day [14], which suggests a large discrepancy between the PA of Swedish boys and girls—the latter being far less physically active than the former. Furthermore, it appears that the number of boys and girls who meet PA recommendations decreases with age [14]. Consequently, to understand the views of adolescent boys and girls from multicultural communities of low SES concerning PA, it is vital to listen to how they express their own meanings of and experiences with PA. In doing so, the identification of facilitators could shed light on ways to create more effective means to increase and maintain the PA of adolescents [3], as well as to stem, if not reverse, the decline in their PA as they grow older. Accordingly, the purpose of this study is to illuminate what adolescents in a multicultural community of low socioeconomic status (SES) in Sweden convey concerning facilitators of PA.

### Theoretical Frameworks

The United Nations Convention on the Rights of the Child emphasizes that every child has the right to express his or her opinion and be heard in all matters affecting his or her health and well-being [15]. In observance, this study is founded upon the perspective of children, which, in this case, means considering both the children’s perspective and a child perspective [16]. On the one hand, the children’s perspective, at least in this study, means listening to voices of adolescents [16] concerning facilitators of PA, which is crucial to advance the understanding of their perspectives on PA (cf. [3]). On the other, a child perspective requires the researchers to interpret the voices of adolescents [16], in this case, through the lens of self-determination theory (SDT), to gain a clearer understanding of their perspectives. This step is essential, for the social conditions in which adolescents develop and function can either thwart or facilitate their processes of self-motivation and healthy psychological development [17].

#### Self-Determination Theory

According to SDT, humans have three innate psychological needs—autonomy, competence, and relatedness—that are essential to their psychological functioning, including mental development, integrity, autonomous motivation, and well-being [18,19]. Autonomy refers to the capacity to control one’s actions and participate in self-chosen activities, whereas competence refers to the feeling of effectively mastering challenging tasks and exercising personal capacity within a given domain. By contrast, relatedness refers to the perception of having meaningful connections with others and feeling comfortable and involved in a given context [18]. SDT also stipulates three dimensions of social environments that can either support or thwart the satisfaction of those innate psychological needs: an autonomy-supportive environment supports the need for autonomy, a well-structured environment supports the need for competence, and an environment with high interpersonal involvement supports the need for relatedness [19]. In particular, autonomy support involves providing meaningful rationales, acknowledging negative feelings, using non-controlling language, offering choice, and nurturing inner motivational resources [20]. Structured environments are predictable, contingent, and consistent [21]. Lastly, interpersonal involvement includes the provision of affection, warmth, care, and nurturance [21]. At the same time, SDT maintains that the development and persistence of a behavior depend on the quality of the motivation [17]. Accordingly, SDT differentiates between controlling motivation (i.e., amotivation, external, and introjected regulation) and autonomous motivation (i.e., identified, integrated, and intrinsic regulation) [17,18]. On the one hand, when an individual is driven by controlling motivation, the behavior is perceived to be controlled and spurred by external factors. One the other, when an individual is driven by autonomous motivation, the behavior is more self-determined and internalized (see [17,18] for greater detail).

## 2. Materials and Methods

By embracing an interpretive approach, this study was based upon the notion that the always-constructed and contextual experiences of adolescents are complex and accommodate shared realities [22]. To capture the nature of these complex, shared realities of adolescents, the study employed focus group interviews, which allowed adolescents to make their voices heard and express themselves freely [23].

### 2.1. Recruitment and Participants

The second-largest city in Sweden, Gothenburg, has experienced widespread socioeconomic segregation, and the district of Angered is one of the most segregated. According to Swedish standards, Angered is characterized by a high proportion of non-native residents, long-term unemployment, low educational level and average income, the need for long-term financial assistance, and poor life expectancy [24]. Angered includes a school attended by roughly 450 students in the fourth to ninth grades that, by Swedish standards, is characterized by a large share of newly arrived students and students of a foreign background [25]. Moreover, the educational achievement scores and the number of students in the ninth grade who pass all subjects are below average [25]. For a more detailed description regarding the school’s characteristics, see the work of Jonsson et al. [26].

In August 2014, all seventh graders at the mentioned school were recruited for the How to Act? research project, an empowerment-based, health-promoting school intervention focused on food and PA habits. Between September and October 2014, all adolescents (12–13 years of age) enrolled in the project were invited to participate in the present study. All 54 adolescents agreed to participate; however, one student was absent during his scheduled focus group, which resulted in a sample of 53 adolescents (21 boys and 32 girls). Prior to enrollment in the project, both the adolescents and their parents or legal guardians received information regarding the project, including its purpose, that participation was voluntary, and that they could leave the study at any time, and the adolescents and their parents or legal guardians provided the researchers with their written informed consent. The project and the present study were approved by the regional ethical review board (Dnr: 469-14).

### 2.2. Procedure and Data Production

To produce data for the study, 10 focus group interviews were performed, each attended by 4 to 6 adolescents; 4 were conducted with girls only, 3 with boys only, and 3 with both genders. The interviews lasted for an average of 69 min (range: 44–97 min). The procedures of the focus group interviews appear in greater detail elsewhere [26].

### 2.3. Data Analysis 

Qualitative content analysis [27,28] was considered suitable to thematize the data in order to obtain a description of the facilitators related to PA expressed by the adolescents. First, focus group interviews were transcribed verbatim, and the transcripts were read several times to “obtain a sense of the whole” [27] (p. 108) and gain a general understanding of the adolescents’ perspectives. Second, a back-and-forth process with decontextualization and contextualization was undertaken to detect similarities and differences in the data. The transcripts were divided into meaning units and sometimes into condensed meaning units, after which the condensed meaning units were abstracted and coded (Table 1). To arrange the codes into tentative categories, similarities and differences between the codes were sorted and compared. The tentative categories were discussed and reviewed several times by the researchers until they were encoded into final categories. In this stage, the analysis concentrated on the visible and obvious parts of the transcripts, meaning that the analysis related to the children’s perspective [16]. Third, adhering to a child perspective [16], the researchers moved back-and-forth between the data and literature to identify appropriate theories to gain a deeper understanding of and support for our interpretation of the adolescents’ perspectives. Fourth, data were compared and contrasted in terms of SDT. In this step, the categories were also arranged into final themes. Although the analysis was performed by the first and last authors, all steps were regularly discussed among the researchers to reach a consensus. The researchers who participated in the analysis represented an interdisciplinary group with broad knowledge of sport science, food and nutrition, and health promotion.

## 3. Results and Discussion 

### 3.1. The Voices of Adolescents Regarding Physical Activity in Genera

To understand what facilitates the PA of adolescents, it is necessary to recognize how adolescents talk about PA in general. When the adolescents in the study discussed PA, they stated that PA need not be exercise or organized sports, but can even be active commuting to and from school or going for a walk with friends. Some of the adolescents mentioned organized PA (e.g., football, martial arts) as a leisure-time activity, and organized PA in terms of physical education (PE), although the adolescents more often highlighted spontaneous PA (e.g., playing unorganized football and basketball with friends) as a common leisure activity (the following quotations illustrates if it is a boy (b) or a girl (g), using pseudonyms, and from which focus group (FG) the quotation is retrieved (e.g., (FG 1)), as exemplified by one of the adolescents: 

Interviewer: Do they usually play football there?

Anna (g): They’re always, you know, at the playground. Just go there, and you’ll see everyone you know. And it’s always football, so you can just go there and play. (FG 1)

Another spontaneous form of PA that the adolescents underscored was playing games outdoors (e.g., hide-and-seek, tag), as shown below:

Interviewer: Do you play any games [outdoors]?

Malik (b): Yes, cops and robbers!

Interviewer: Cops and robbers?

Malik (b): Yes! And hide-and-seek.

Caleb (b): Zombie tag. (FG 4)

Although spontaneous PA has decreased in Sweden significantly during the last few decades [14], spontaneous PA seems to be an essential part of these adolescents’ accumulated PA, as for among other adolescents from families of low SES (cf. [29]). Moreover, Fröberg et al. [30] conducted a quantitative study with this sample, and data extracted from that study showed that only 32% of the participants in this study were active in sports clubs, compared to another report, which suggested that about 64% of Swedish adolescents are members of sports clubs [14]. As such, the adolescents in this study appeared to be less physically active in sports clubs compared to children and adolescents in Sweden in general, although participation in sports clubs is considered an important part of youths’ leisure and everyday life, as it is regarded to be beneficial to democracy, equality, and public health [31]. As shown by Jonsson et al. [26], the lack of participation in sports clubs among these adolescents may relate to lacking parental and financial support. Consequently, it is important that the neighborhood area where these adolescents often socialize is designed so that they are encouraged to engage in spontaneous PA.

In response to the study’s aim, two major themes were identified—one was the possibility for enjoyment, and the other was social support and a supportive environment—as well as six categories, all of which illuminate facilitators concerning the adolescents’ PA (Table 2). In what follows, the categories are described and illustrated with quotations that are characteristic for each category. Each category begins by illuminating the adolescents’ voices, and is followed by an interpretation of the finding in relation to previous research and, if suitable, through the lens of SDT.

### 3.2. Possibility for Enjoyment 

#### 3.2.1. Personal Preference and Fun Guides Physical Activity Engagement

The adolescents stated that for them to engage in PA, the PA should be fun. What constituted fun PAs varied, however, and as did the activities that the adolescents mentioned (e.g., playing games, football, basketball, martial arts), as illustrated below: 

Interviewer: How come you play football then?

Zavier (b): Because it’s fun. 

Jamel (b): Because it’s fun. (FG 7)

Overall, the findings are consistent the results of previous studies, which have highlighted the need for adolescents’ PA to be fun [32,33], and that fun relates closely to intrinsic motivation [18]. For instance, a systematic review and meta-analysis by Owen et al. [34] showed that autonomous motivation (i.e., intrinsic and identified regulation) was positively associated with children’s and adolescents’ PA levels, which highlights the importance for adolescents to engage in PAs that they find personally meaningful and enjoyable. 

In some of the focus groups, the adolescents mentioned that boys, in general, are more physically active compared to girls. In other focus groups, however, the adolescents described different views on whether boys are more physically active than girls. The adolescents’ reasoning ended with the idea that PA engagement depends more on personality and personal preferences, as illustrated below: 

Interviewer: Are there any differences between boys’ and girls’ leisure time activities?

Hamza (b): Boys usually participate more in sports.

Interviewer: More than girls?

Amarion (b): No, not how I see it.

Interviewer: What do you think?

Amarion (b): I don’t want to distinguish between the sexes. There are girls who play a lot of sports, and there are guys who are not so much into sports. So, it depends on how you are as a person.

Interviewer: It’s not about sex, but about who you are and what you like?

Mina (g): Yes, what you like. (FG 9)

However, research has shown that boys are generally more physically active and participate more in sports clubs than girls [14], and in the present study’s sample, only 24% of boys and 6% of girls were physically active enough to meet current PA recommendations [30]. Moreover, when discussing which sports clubs the adolescents attended, one boy emphasized that dance was something that he liked, as shown below:

Ahmad (b): And dance. Albanian dance.

[The other adolescents burst into laughter.]

Ahmad (b): What? I like it! (FG 4)

Leisure time activities such as dance are commonly viewed as being feminine [35], and it is possible that boys in the above-quoted group shared that view, which could explain their laughter. From a SDT viewpoint, however, the boy who liked to dance took the opportunity to express his volition and, as such, satisfy his need for autonomy (cf. [18]).

#### 3.2.2. Variation Makes Physical Activity Fun

The adolescents suggested that it is essential to try different activities until one finds activities that he or she personally enjoys. They also mentioned variation as a way of making PA fun. For example, the adolescents highlighted that they liked PE because it provided them with the opportunity of choosing and trying several different PAs. Traditional teaching in PE supplies pupils with a version of “sports light” [36], and this kind of variation in PA makes PE fun, as exemplified in one of the focus groups:

Interviewer: How come you like it [PE]?

Grace (g): Because you try different activities. You don’t do the same thing every lesson. (FG 5)

Similarly, in relation to organized sports in sports clubs, the adolescents suggested that it is important to try different sports until you find one that you like, as illustrated by of one of the adolescents:

Jazmin (g): You can try different ones, until you find a sport that you like. (FG 3)

In a similar vein, Martins et al. [3] showed that autonomy is important in relation to adolescents’ PA engagement. Variation and the opportunity to choose from among different forms of PA relate to the basic need of autonomy [18]. Because SDT stipulates that satisfying the need for autonomy promotes intrinsic motivation [18], it makes sense that variation and the opportunity to choose among different PAs promoted the adolescents’ perceptions of fun.

#### 3.2.3. Physical Skills Make Physical Activity Fun

Some of the adolescents mentioned that PA was especially fun when they felt skilled and competent at the activity, which was mentioned in relation to PE, spontaneous PA, and participation in sports clubs. The adolescents stated that they felt competent when they learned new skills and developed their physical abilities. Similarly, some of the adolescent girls mentioned that they liked to play basketball in PE because they were good at it, as seen below:

Interviewer: Is there something else that you would like to do in physical education that you think is more fun?

Kayla (g): No, not really. We girls like to play anything, as long as we’re good at it.

Sara (g): Basketball.

Kayla (g): Yes, girls like to play basketball because they’re skilled at it. (FG 2)

When the adolescents discussed feeling competent, they neither mentioned winning nor focused on performance. Instead, they emphasized the importance of doing their best and having fun, as exemplified in one of the focus groups:

Interviewer: Is it important to win?

Filip (b): No, not as long as you play well. 

Malik (b): And have fun.

Filip (b): Then, it’s not important to win. 

Malik (b): As long as you have fun. (FG 4)

It is therefore problematic that organized PA, in sports clubs, usually focuses on performance-based results with a win-at-all-costs mentality, and encourages competitive pressure (cf. [37,38]) instead of emphasizing doing one’s best and the fun of the activities. In a similar vein, Martins et al. [3] observed that adolescents characterized a competitive, performance-oriented environment by a lack of fun. Other studies have highlighted that feelings of competence contribute to the experience of fun related to adolescents’ PA [32]. For instance, Martins et al. [3] also found that perceived competence was an important factor for adolescents’ PA maintenance, whereas a lack of competence decreased adolescents’ PA over time [3]. From an SDT-based perspective, it is logical that feelings of competence make PA fun, for SDT stipulates that satisfying the need for competence promotes intrinsic motivation [17].

#### 3.2.4. Friends Make Physical Activity Fun

The adolescents mentioned that being physically active together with friends contributed to their sense of fun during PA. Social interaction as an important motivational factor was exemplified in one of the focus groups, as follows: 

Interviewer: How is it that you engage in sports?

Caleb (b): To have fun and spend time with friends.

Jamal (b): You can hang out with your friends.

Malik (b): You’re with friends. You play together, you swim together, and you wrestle together. (FG 4)

This finding aligns with the results of several previous studies that have shown that friends are an important part of adolescents’ engagement in PA, and that being physically active with friends makes PA fun [32,33]. Being physically active with friends is closely linked to the need for relatedness (cf. [17]). According to Ryan and Deci [18], satisfying the need for autonomy and competence promotes intrinsic motivation, whereas satisfying the need for relatedness promotes intrinsic motivation only in a more distal sense. By contrast, several quantitative studies have failed to identify a link between adolescents’ satisfaction of the need for relatedness and autonomous motivation in PA contexts and PE (e.g., [39]). The present study, however, found support for the notion that satisfying the need for relatedness is important for adolescents’ intrinsic motivation in PA contexts.

Altogether, this theme illustrates that professionals (e.g., teachers, school nurses, health pedagogies, coaches) who work with adolescents should create opportunities for adolescents to engage in PA that they find fun and enjoyable, while also seeking to reduce the competitive and performance-based nature of PA. According to the adolescents, fun and enjoyable PAs offer options and variation (e.g., different activities, options in relation to each activity), opportunities to learn and develop (e.g., new skills, progress in activities that develops existing abilities), and comradery (e.g., activities with friends or classmates). From a SDT-based perspective, it is essential that adolescents’ PA occurs in an environment that supports their need for autonomy, competence, and relatedness (cf. [18,19]). It is therefore crucial that professionals in PA contexts provide adolescents with autonomy support, structure, and interpersonal involvement (cf. [19]).

### 3.3. Social Support and a Supportive Environment

#### 3.3.1. Encouragement from Family and Having Friends to Be Physically Active with

When the adolescents discussed whether they engaged in organized PA, they mentioned their parents’ support as an essential factor that, without which, would make it difficult for them to participate in organized PA. The adolescents also expressed a wish for their family members to be even more supportive of their PA by, for example, engaging in PA together with them or showing that they cared about their PA, as exemplified by one of the adolescents:

Interviewer: What would it take for you to engage in some (physical) activities?

Yesenia (g): That you get some help with it. If you’d like to start something, then that someone shows that they care about it. You could tell your parents what you’d like to do. And then they could help you with it. (FG 1)

Moreover, the adolescents reported that it is easier to be physically active with friends. If they were to join a sports club, for instance, then they stated that they would feel safer if a friend joined as well. They also mentioned that if friends had tried a sport, then they could ask them how it was, which could pique their own interest in trying it. Arguably, if adolescents are surrounded by friends who are members of sports clubs, then that circumstance could increase the odds of them also joining the sports club. As one adolescent explained, it is also easier to be physically active somewhere other than home, especially with friends: 

Yesenia (g): I think it’s much better to train somewhere other than home, because if you do it at home, then you might skip a few workouts sometimes, but if you do it with someone else, you have to do it, you know. If you’re working out with someone else, you have to, like, get up and do it. (FG 1)

Overall, these results are consistent with the findings of previous research, which have shown that social support from family and friends is associated with adolescents’ PA levels [4]. The quotation can be interpreted to mean that being physically active with friends creates positive peer pressure, as adolescents do not want to disappoint their friends by refraining from PA. Presumably, adolescents are, to some extent, driven by introjected regulation (i.e., guilt or anxiety avoidance) to be physically active [18]. Previously, introjected regulation has been both positively [40] and negatively [34] associated with adolescents’ PA. Although introjected regulation and other controlled forms of motivation have been associated with maladaptive psychological functioning [18], they can serve as the first step toward internalizing a certain behavior [41], including PA behavior. Lastly, social support from family and friends can interpreted as being similar to what SDT terms “interpersonal involvement” [21], which research has shown to be important for satisfying the need for relatedness [19]. As such, social support provided by adolescents’ family and friends arguably facilitates the adolescents’ feelings of relatedness. Satisfying the need for relatedness has been shown to increase the likelihood of internalizing a behavior [18], and it therefore makes sense that social support from family facilitates adolescents’ PA.

#### 3.3.2. A Supportive School Environment

The adolescents had several suggestions for how the school environment could become more supportive of their PA. Some mentioned that they would like longer breaks at school so that they could have more time to go outdoors and be physically active, as illustrated below:

Interviewer: What would you do if you had longer breaks?

Kayla (g): We’d go outside.

Sara (g): Nothing.

Kayla (g): We’d, you know… we’d go outside—at least I would.

Naima (g): I would, too. 

Layla (g): We’d go out and chase each other, play hide-and-seek. (FG 2)

In a similar vein, children in Pawlowski et al.’s [42] study identified the short duration of school breaks as serving as a barrier to being physically active. Moreover, some of the adolescents in this study stated that they would have liked more PE lessons per week, whilst other adolescents said that they would have liked the PE lessons to have been longer. PE in elementary school is intensively debated in Sweden at the moment, as the Swedish government recently proposed that from the beginning of 2019, PE in elementary school would receive 100 more hours of teaching [43]. Lastly, in relation to PE, the adolescents declared that they desired more influence over the PE lessons and the opportunity to decide upon which activities to engage in during the PE lessons. Hence, this could be interpreted as the adolescents wishing that their PE teachers were more autonomy supportive (cf. [44]) and provided them with more options during PE.

Overall, our findings for the theme demonstrate that professionals working with adolescents in PA contexts could explore adolescents’ sources of and opportunities for social support, as well as encourage adolescents to seek support from family and friends. Because social support from family members is essential for adolescents’ engagement in PA [4], it is important that family members act as good role models in relation to PA, and engage in PA with adolescents in their families as much as possible. Of course, it could be difficult for unaccompanied refugee children, for instance, to experience social support from their families related to PA, or for single parents to provide their children with sufficient support in PA. As Jonsson et al. [26] have shown, because adolescent girls of some cultural backgrounds are expected to engage in household chores instead of PA, their family members might be unwilling to support their engagement in PA. Consequently, it becomes even more important to address the adolescents’ suggestions regarding how their school could better support their PA, including by offering longer breaks and more PE lessons. It is thus worth considering organizational structures at schools and, if possible, extending the duration of breaks, devoting more hours in the curriculum to PE, and organizing activities before or after the school day to offer adolescents the opportunity to be physically active.

**Figure 1:**
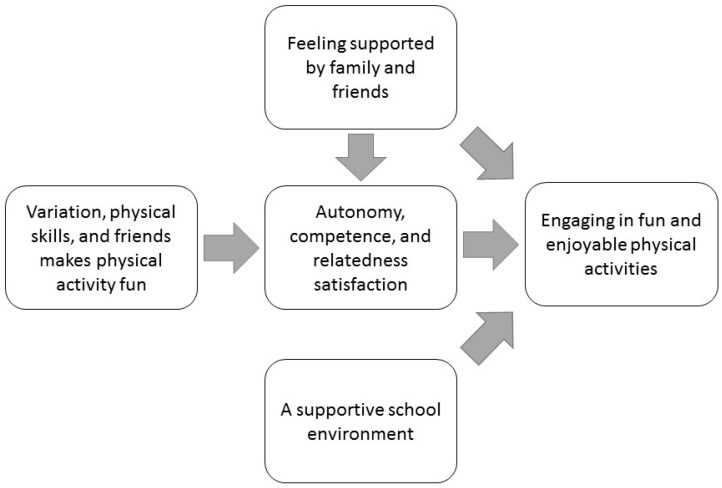
Empirical model that summarizes the study’s findings, based on the adolescents’ voices and the researchers’ interpretation of the empirical data through self-determination theory (SDT).

The facilitators of PA highlighted by the adolescents can be summarized as illustrated in Figure 1. For one, when the adolescents discussed PA, they emphasized the need for it to be fun and enjoyable for them to want to participate in the first place. For adolescents, engaging in a variety of PAs and having options, learning new skills, developing abilities in PA contexts, and being physically active with friends contributes to the fun and enjoyableness of PA. Arguably, engaging in a variety of PAs and having options facilitates the satisfaction of the need for autonomy, learning new skills and developing abilities in PA contexts promotes the satisfaction of the need for competence, and being physically active with friends and feeling supported by family and friends stimulates the satisfaction of the need for relatedness. Indeed, as SDT maintains, satisfying basic needs promotes intrinsic motivation [18,19]. Moreover, according to the adolescents, social support from family and friends facilitated their PA engagement, and they had several suggestions regarding how the school environment could facilitate their engagement in PA by, for instance, extending breaks and offering more PE lessons.

## 4. Method Discussion

Focus group interviews allow the participants to make their voices heard and to express themselves freely [23]; hence, it can be argued that the use of focus groups aligns with The United Nations Convention on the Rights of the Child [15]. Conducting research interviews, however, is accompanied by power relations [45,46], and because three adults were present during each focus group, a great power imbalance may have been present. The power imbalance may have inhibited the adolescents’ opportunity to express themselves freely, or may have contributed to the adolescents feeling inclined to answer the questions in a certain way. In trying to reduce these power imbalances, we aimed to develop trust between the interviewers and the adolescents by, for example, sharing something personal about ourselves. Additionally, in the beginning of the focus groups, we explained that all statements would remain within the group, that all thoughts and ideas were important, and that there were no right or wrong answers.

As the literature specifies [23], we aimed at keeping boys and girls in separate focus groups for practical reasons (e.g., school timetable and wishes from the classroom teachers); however, it was not possible to keep boys and girls separate for all the focus groups. Hence, in the mixed-gender focus groups, some of the adolescents may have felt inhibited to speak freely. A limitation with the study was that we did not collect any additional background information, other than gender and age, on the participants. Another limitation with this study was that a few of the adolescents did not speak Swedish fluently, which prevented them from expressing themselves freely. Hence, in retrospect, an interpreter would have been of good use. Because all the adolescents included in this study participated in regular teaching (in Swedish), however, we were under the impression that an interpreter was not necessary.

For a comprehensive discussion regarding the trustworthiness of the study findings, see Jonsson et al. [26].

## 5. Conclusions

When the adolescents discussed PA, they mostly referred to spontaneous PA in the neighborhood (e.g., playing football with friends, playing tag) instead of organized PA (e.g., being active in a sports club). To the best of our knowledge, the importance of spontaneous PA seems to be specific to the sample, as spontaneous PA has decreased significantly over the last few decades among children and adolescents in Sweden, in general. Arguably, spontaneous PA thus facilitates the adolescents’ daily accumulated PA. As such, it is essential that the neighborhood area where the adolescents often socialize is designed to encourage spontaneous PA (e.g., through availability of green spaces and sports grounds). The adolescents highlighted the need for PA to be fun and enjoyable and without a competitive or performance-based orientation. For them, PA was fun and enjoyable when they engaged in a variety of forms of and had options for PA, learned and developed new skills in PA contexts, and were physically active with friends. They also mentioned that social support from family and friends facilitated their PA engagement, and they had several suggestions regarding how the school environment could better support their PA (e.g., with longer breaks and more PE lessons). From an SDT-based perspective, it seems important to facilitate adolescents’ intrinsic motivation with a supportive PA environment that satisfies adolescents’ need for autonomy, competence, and relatedness. Because the adolescents highlighted that PA should be fun and enjoyable and without a competitive or performance-based orientation, for future directions, it might be of interest to employ a similar approach as this study among adolescent sports club members.

## Figures and Tables

**Table 1:** Examples of meaning units, codes, categories, and themes from the data analysis.

Meaning Unit	Code	Category	Theme
To have fun and spend time with friends	Fun with friends	Friends make PA fun	Possibility for enjoyment
It depends on whether your family supports you. If you say that you would like to start playing football, and they say no, then it becomes difficult because your family does not support you.	Support from family	Encouragement from family and having friends to be physically active with	Social support and a supportive environment

PA: physical activity.

**Table 2:** Categories and themes regarding facilitators of the adolescents’ physical activity (PA).

Category	Theme
Personal preference and fun guides PA engagement	Possibility for enjoyment
Variation makes PA fun	
Physical skills make PA fun	
Friends make PA fun	
Encouragement from family and having friends to be physically active with	Social support and a supportive environment
A supportive school environment	
